# Life Saving in the London Streets

**Published:** 1901-12-28

**Authors:** 


### The Institutional Workshop.

### LIFE SAVING IN THE LONDON STREETS.

It is a mistake to say that London has done and is doing nothing in regard to life-saving in the streets. It is to the credit of Englishmen that much of the good work accomplished by voluntary effort should be done so quietly and anonymously that, as in the present case, London has a fairly complete system of street ambulances which have been rendering efficient service for ten years, although the majority of the citizens of London are unaware of the fact. Prior to the establishment of the Hospitals Association Street Ambulances Service at the end of 1889, cabs were almost exclusively employed in the transport of cases of street accident, with the well-known result of frequent conversion of slight casualties to grave injuries. During the first three years of its existence this service superseded by its ambulances the cab as a means of transport for cases of accident in one out of every three cases. In round figures, 3,400 cases were removed on street ambulances in the first three years, an average of 1,133 cases per annum. Year by year the number of accidents occurring in the streets which have been handled by the street ambulance service has increased, and at the present time from two-fifths to one-half of the whole number of accidents occurring in the London streets are, there is good reason to believe, dealt with by this service. It follows that it is not only not correct to state no scheme has been propounded for London, but, in fact, a regular service, as I will proceed to show, is actively at work in our midst, thanks to the generosity of one of its citizens, Mr. H. L. Bischoffsheim.

### History of the Movement in London.

Prior to the year 1889 the organisation of a complete ambulance service for the whole of the metropolis had never been even attempted, in a practical manner. So long ago as 1860, that is, before the word ambulance was coined, the late Mr. W. J. Nixon, then house governor of the London Hospital, with others, took part in a movement for supplying carriages for the removal of infectious cases. This, the first project of the kind, was confined to infectious cases only, and so recently as 1890 the London Hospital had still one of these carriages connected with it. Some years later, under the presidency of the Duke of Cambridge, a scheme was proposed to make the hospitals the ambulance stations, and to provide for the transport to hospitals of persons injured in the street. Each ambulance was to be drawn by a pair of horses, and suitable accommodation for the horses and ambulances, with the necessary funds, were, it was suggested, to be provided by the hospitals themselves out of their resources. It is not surprising that this scheme was a failure. At a meeting of the Hospitals Association in April, 1885, Captain William Joynson, chairman of the Northern Hospital, Liverpool, read a paper on horse ambulances in connection with the hospitals, in which he described the service established in 1883 in connection with his hospital. That service was an adaptation of the New York system, the characteristic features of which are that the hospitals are the ambulance depôts and the police stations the call offices, the whole of them being in telegraphic or telephonic communication with the hospitals. The speakers at that meeting referred to the absence of ambulance provision in London, and all expressed the view that how to deal with it was an extremely difficult question. A strong committee was then appointed to find a practical solution, but although the St. John's Ambulance Association was represented on that committee, it resulted in nothing. In 1882 something was done for one locality, Kilburn, a district where there was not a great traffic, and then a somewhat remote part of the Metropolis, though just within the four-mile circle. It commenced with one litter station, and after four years another was established at a short distance from it, and round these two stations as a centre were placed at distances averaging from one-half to three-quarters of a mile six stretcher stations. In case of accident the nearest stretcher was sent for, and a messenger was also dispatched to one of the two central stations for the wheeled litter. By the time the injured person had been attended to and placed on the stretcher, this litter was on its way. The sufferer was carried to meet it; the stretcher was fitted into its place, and the patient was then quickly wheeled to the hospital. This Kilburn service was supported by public subscription, and was the only attempt at an ambulance organisation for London, until the establishment of the street ambulance service of The Hospitals Association in 1889. About the same time the St. John's Ambulance Association were enabled by the generosity of Dr. Freshfield, who defrayed the whole expense, to establish an excellent station under the west portico of St. Paul's Cathedral; and the same Association placed a stretcher at No. 3 St. Bride Street, near Ludgate Circus. The authorities of King's College Hospital had also obtained an ambulance for use in connection with their institution. Such were the ambulance arrangements of London when the present street ambulance service was organised.

### The Present Street Ambulance Service.

This service was the outcome of a scheme for the organisation and maintenance of an effective street ambulance service for the metropolis laid before a meeting of The Hospitals Association on March 20th, 1889, by Mr. Thomas Ryan. The scheme was unanimously adopted by the Association, was warmly advocated by the press, and met with such prompt support that the funds necessary for its establishment and maintenance for one year were immediately subscribed. Mr. H. L. Bischoffsheim, the present treasurer, has from the outset taken the warmest interest in this movement, and he has very generously provided the necessary funds for its maintenance year by year, the total cost having so far exceeded £5,000. To Mr. Bischoffsheim for his great generosity, and to Mr. Thomas Ryan who has managed the service from the outset, the grateful thanks of all Londoners are due. The first step taken was to subdivide London into districts, and, the object being to help the police as much as possible and to work in conjunction with them, it was decided to adopt as the ambulance districts the divisions of the Metropolitan Police. These districts were marked off on the ordnance survey map of London, on which was shown conspicuously all the police stations, fire brigade stations, hospitals, and railway stations within the four-mile circle. It was attempted, with a large measure of success, to secure accommodation for the ambulances in suitable situations free of all cost by obtaining the co-operation of the authorities in charge of police stations, fire brigade stations, hospitals, railway stations, and other public buildings. Accommodation was freely placed at the disposal of the ambulance committee at all these places, with the exception of police stations, an exception which was more than made good by the police themselves, who suggested in the first place and promptly carried the suggestion into practice that cab ranks would be the most appropriate places at which to establish ambulance stations. Mr. Ryan insisted that "an essential condition to be observed in any proposals for perfecting the ambulance system is, that no additional work or responsibility shall be cast upon the police. So far from this objection attaching to the street ambulance system, the direct opposite is the case. The responsibility of the police is not affected, and, as regards the amount of work they are required to do, the scheme multiplies the appliances for its performance and places them in the most accessible positions for use. In other words, with or without a street ambulance service the character and extent of the work of each policeman remains the same; but this service, by providing instruments for its performance, has in fact very largely reduced the labour of the police authorities. The Chief Commissioner of the Police warmly co-operated, and with their assistance ambulances were established in close juxtaposition to most of the busiest thoroughfares.

### Street Ambulance Stations and Methods.

At the present time there are between 50 and 60 street ambulance stations within the four-mile radius. Of these, 10 are fire-brigade stations, 12 are hospital stations, and 26 are thoroughfares, like the Marble Arch, Blackfriars Bridge, Royal Exchange, Holborn Town Hall, Langham Place, Rye Lane, Peckham, All Saints' Church, Poplar, Elephant and Castle, and the Holborn Viaduct. The statistics of the work done in 10 years, 1891–1900, show that the ambulances have been employed from the date of the establishment of the service some 17,775 times. During last year 250 accidents were dealt with at the fire-brigade stations, 834 at the hospital stations, and 1,320 at the thoroughfare and other stations, making a total of 2,404 accident cases. It is matter for regret that these figures are largely approximate only, owing to the difficulty of keeping precise records at each ambulance station in existing circumstances. In the case of the fire-brigade stations, however, a careful record is kept of each use of the ambulance, and the same is done by the City police with regard to the ambulance at the Royal Exchange, whilst several hospitals compile a return of the work done each year by their ambulance. I am content, however, to quote the precise figures of the fire-brigade stations as showing the continuous growth and the usefulness of the street ambulance service. In 1891–2 96 accident cases were removed from fire-brigade stations, and the annual number had increased to 250 according to last year's return. Here I would bring out a useful point, which is not appreciated by those who have no practical experience of ambulance work. The amount of mischief an injured person is likly to sustain through being removed by an unskilled person from the spot where he has fallen to a stretcher placed at his side is as nothing to the injury he will suffer in being lifted into and extracted from a four-wheeled cab or a hansom by the most skilful bearer that ever was trained. Nor is this all; for the effects of the journey in a sitting posture in a shaky cab compared with the results of conveyance, lying at full length on a stretcher in a litter with indiarubber tired wheels, all will be able to appreciate. Whether, therefore, London ultimately has a horse ambulance or not, it would seem to be essential that the present street ambulance system should be maintained and extended, so as to secure that litters and stretchers shall be available near every crowded thoroughfare to receive the accident cases pending the arrival of the horse or electric motor ambulance. I may note further that the pattern of street ambulance at present in use is the one selected as the result of a public exhibition and competition, when the judges appointed were eminent members of the medical profession, with special knowledge of ambulance work. Attached to each street ambulance is a basket containing splints, bandages, and other requisites for rendering first aid to the sufferer, and an inspector has been appointed whose duty it is to make a continuous round of the stations, to clean and lubricate the ambulances and keep them in good repair and thorough working order. Finally, according to the report of the police commissioners, the number of accidents known to the police in London each year is about 6,000, and of these upwards of 2,400 were removed by the street ambulances last year. It is matter for wonder, in view of the fact that an ambulance is stationed on the Embankment at Blackfriars Bridge, that it should have been possible for a case of neglect like that which was recently reported in a daily paper to have occurred at all.

### The Police the Key to the Problem.

In London the police hold the key to the wise solution of the ambulance difficulty. The policeman's instructions are to keep the roads clear for the traffic; that is their first duty, and as a consequence, and for reasons I will mention, there is ground for fearing that many accident cases are still placed in cabs by the police, which should be placed upon the adjacent ambulance. The explanation of this is that it is simpler and less troublesome to whistle up a cab than to go to the adjacent ambulance station, take out the ambulance, and then proceed with the patient to the hospital. A further difficulty arises from the fact that many of the police have not received instruction in first aid. Much, no doubt, has been done in this direction in recent years, but until arrangements are made to enable the police to be instructed in first aid during a portion of their duty time, it is not reasonable to hope that the whole of the men engaged will have sufficient public spirit to secure instruction in first aid by giving up a portion of their day when off duty. It is not fair to the public or the police that this should be expected of the men, and as matters stand at present, unless I am greatly misinformed, the attitude of the authorities seems to display much less public spirit than that expected from the policeman, who voluntarily undergoes a course of such instruction. Again, I think the use of the street ambulance service would be much extended, and it would be juster to the police, if the Chief Commissioner could see his way to permit a fee of, say, half-a-crown to be paid to each policeman who conveyed an accident case to the hospital on a street ambulance. In saying this I desire to make it perfectly clear that most of the success achieved by our present voluntary system of street ambulances is due to the co-operation of the Chief Commissioner and the men under him, and no one who is much in the streets of London, as I am, can fail to feel grateful to our police force for the really excellent service which they render to Londoners at all times.

### Horse or Electric Motor Ambulances.

A gentleman who desires to remain anonymous has often told me that if this kind of ambulance can be usefully established in London, he would defray the initial outlay, providing arrangements could be made to supply stations and for the maintenance of an efficient service. My answer has been that the circumstances of London differ so greatly from most great cities abroad and in the United States that I have grave doubts as to the suitability of the American system for the metropolis. As Mr. Ryan and others have pointed out, there are four main objections to the adoption of such a system: (1) The London hospitals are so unevenly distributed over the metropolis, all but two of them being on the north side of the river, and the majority situated close together around Charing Cross, that they are most unfavourably placed for becoming effective ambulance stations. (2) The London hospitals, or the majority of them, have neither the space to devote to the stables and other accommodation required for a motor or horse ambulance station, nor the means to maintain it; and if they had a surplus of both they would need to expend it in advancing their recognised work in preference to employing it to establish a new departure which would greatly extend their responsibilities. (3) So enormous a place as London, with accidents happening simultaneously in the same district in different quarters could be more effectively ambulanced by a large number of hand litters stationed at short distances apart, all over the area, than by a limited number of horse or electric-motor ambulances. (4) In Continental and American communities the municipalities or the State expend large sums out of the rates and taxes on various organisations, including the ambulance service, and until the citizens are prepared to face much higher rates than they have to pay at present, it seems hopeless to expect that so expensive an organisation as an electric-motor ambulance system for the whole of the metropolis, with a large paid staff of medical men and attendants, will be provided promptly at the public expense. The difficulty of medical service in London would be great, apart from the expense altogether, and the end of the whole matter, so far as I can see, is to advocate and support the extension of the existing street ambulance service by improving its efficiency, extending the number of its stations, and possibly supplementing the present hand ambulances by one or two electric motor ambulances for experimental purposes in dealing with the cases in the more crowded centres. In this connection it is interesting to note that the municipality of Battersea has intimated to the street ambulance association that they have determined to place a large number of st eet ambulances throughout their area, and to maintain them at the public expense. What Battersea has done other metropolitan boroughs may do, and I think it is greatly to its honour that so relatively poor a district as Battersea, crowded as it is with the humbler citizens, should have displayed this amount of public spirit and public intelligence. H. C. B.

